# Simultaneous infections by different *Salmonella* strains in mesenteric lymph nodes of finishing pigs

**DOI:** 10.1186/1746-6148-10-59

**Published:** 2014-03-07

**Authors:** Victoria Garrido, Samanta Sánchez, Beatriz San Román, Ana Zabalza-Baranguá, Yasmin Díaz-Tendero, Cristina de Frutos, Raúl-Carlos Mainar-Jaime, María-Jesús Grilló

**Affiliations:** 1 Animal Health, Instituto de Agrobiotecnología (CSIC-UPNA-Gobierno de Navarra), Pamplona, Spain; 2 Central Veterinary Laboratory, MAGRAMA, Madrid, Spain; 3 Departamento de Patología Animal, Facultad de Veterinaria, Universidad de Zaragoza, Zaragoza, Spain

**Keywords:** *Salmonella*, Multiple infections, Pigs, Serotypes, Antimicrobial resistance

## Abstract

**Background:**

Salmonellosis is a major worldwide zoonosis, and *Salmonella*-infected finishing pigs are considered one of the major sources of human infections in developed countries. Baseline studies on salmonellosis prevalence in fattening pigs in Europe are based on direct pathogen isolation from mesenteric lymph nodes (MLN). This procedure is considered the most reliable for diagnosing salmonellosis in apparently healthy pigs. The presence of simultaneous infections by different *Salmonella* strains in the same animal has never been reported and could have important epidemiological implications.

**Results:**

Fourteen finishing pigs belonging to 14 farms that showed high salmonellosis prevalence and a variety of circulating *Salmonella* strains, were found infected by *Salmonella* spp, and 7 of them were simultaneously infected with strains of 2 or 3 different serotypes. Typhimurium isolates showing resistance to several antimicrobials and carrying mobile integrons were the most frequently identified in the colonized MLN. Four animals were found infected by *Salmonella* spp. of a single serotype (Rissen or Derby) but showing 2 or 3 different antimicrobial resistance profiles, without evidence of mobile genetic element exchange *in vivo*.

**Conclusion:**

This is the first report clearly demonstrating that pigs naturally infected by *Salmonella* may harbour different *Salmonella* strains simultaneously. This may have implications in the interpretation of results from baseline studies, and also help to better understand human salmonellosis outbreaks and the horizontal transmission of antimicrobial resistance genes.

## Background

Acute gastroenteritis caused by *Salmonella* spp. represents a Public Health concern because of its high welfare and socio-economical impact in developed countries [1,2]. In the USA, salmonellosis is the main cause of foodborne illness with 1,027,561 human cases of non-typhoidal salmonellosis in 2011, of which a total of 19,336 (1.9%) required hospitalization and 378 (1.95%) had a fatal outcome [1]. In the European Union (EU), salmonellosis is, after campylobacteriosis, the most common zoonosis, registering a total of 95,548 human cases in 2011 [3].

Besides laying hens and poultry, asymptomatically *Salmonella*-infected pigs are a major source of human salmonellosis [4-6], by intermittently shedding the pathogen in their faeces and thus contaminating pork and products thereof. However, faecal excretion is not neccesarily indicative of a true infection of the animal. In fact, after being ingested, *Salmonella* may be present in faecal samples and pass through the pig gut lumen without invading the enterocytes. To cause active infection, salmonellae should invade the enterocyte barrier and reach the local lymphoid system [7]. Accordingly, the proper diagnosis of this infection in pigs requires the identification of this pathogen in the mesenteric lymph nodes (MLN). Thus, EU reference studies in finishing pigs have been based on the detection of *Salmonella* spp. in MLN at slaughter.

Pigs are considered susceptible to most of *Salmonella* serotypes and, although Typhimurium is the most common, a large variety of other serotypes are also reported in surveillance studies at farm level [5-8]. However, the presence of multiple infections in MLN of a single animal, although suggested, has never been confirmed.

An additional challenge for human health is the emergence of multi-antimicrobial resistant (AR) *Salmonella* strains and the subsequent spread of the AR clones [9]. Pigs and other domestic species are recognized as a primary reservoir of multi-AR bacteria, usually associated with the selective pressure exerted by antimicrobial treatments [10]. The emergence and spread of multi-AR *Salmonella* are often related to both the acquisition and the fixation of bacterial mobile genetic elements such as plasmids, transposons or integrons [11]. Five classes of integrons carrying antibiotic resistance gene cassettes have been reported so far [12]. Class 1 Integrons (IC1) are the most prevalent in the *Enterobacteriaceae* family, containing different AR gene cassettes (e.g. *pse1* and *aadA2*, characteristic of Typhimurium phage-type DT104) that can be located either extrachromosomally or integrated in the *Salmonella* Genomic Island 1 (SGI1) [13]. Coexistence in the same animal of *Salmonella* strains showing different AR genes has been postulated to support the horizontal AR genetic exchange.

The aim of the present study was to ascertain whether different *Salmonella* serotypes and/or strains can be simultaneously isolated from the same animal.

## Methods

### Experimental design, *Salmonella* spp. isolation and serotyping

A total of 14 fattening pig farms identified previously [8] with high herd *Salmonella*-prevalence and showing multiple circulating strain types (serotypes and/or AR profiles) were selected for this study. One pig from each farm was randomly selected at the slaughter line in the abattoir. Animal handling and slaughtering procedures were performed according to the current national legislation (Law 32/2007, for animal care on holdings, transportation, testing and slaughtering). The whole intestinal package was removed from the selected carcasses at the evisceration point of the slaughter line, and MLN samples (25 grams from at least 5 MLN) were collected in a sterile plastic bag (Stomacher® 80, Seward Medical), transported at 4°C to the laboratory and immediately processed for *Salmonella* isolation. Isolation procedures were performed according to ISO 6579:2002/Amd 1:2007 rules [14], as described previously [8]. After selective growth (37°C, 24 h) on Xylose Lysine Deoxycholate Agar (XLD) and Brilliant Green Agar (BGA) plates, 10 presumptive *Salmonella* spp. colonies from each MLN sample were tranferred from selective plates to agar, then tested biochemically (triple sugar iron, urease agar, indole reaction and L-lysine decarboxylation tests) and further confirmed by serotyping at the National Reference Laboratory Centre for Animal Salmonellosis (Madrid, Spain), following the Kauffmann-White Scheme [15].

### Antimicrobial resistance

A total of 140 *Salmonella* colonies were tested by the Kirby-Bauer disk diffusion method [16] using the antimicrobials and concentrations recommended by the current EU legislation for harmonized monitoring of antimicrobial resistance of *Salmonella* in poultry and pigs [17], namely, Ampicillin and Amoxicillin plus Clavulanic acid (A), Chloramphenicol (C), Streptomycin (S), Gentamicin, Sulfisoxazole and Trimethoprim plus Sulfamethoxazole (Su), Tetracycline (T), Nalidixic acid (Nx), Enrofloxacin, and Cefotaxime (BD Diagnostics). *E. coli* strain ATCC 25922, and serovar Typhimurium strains ATCC 14028 and DT104 were used as controls. Antimicrobial susceptibility was determined by measuring the inhibition halo generated after incubation (37**°**C, 24 h). Strains were classified as resistant or susceptible, according to the Clinical and Laboratory Standard Institute (CLSI) recommendations [18].

The presence of IC1 was analysed by PCR using the primers 5′CS-3′CS described previously [19], and the resulting amplicons were purified with a commercial kit (ATP), cloned in pGEM^®^-T (Promega), and then sequenced (Secugen). DNA sequences were analysed by ExPASy protein translation (SIB Bioinformatics Resource Portal) followed by Protein Basic Local Alignment Search Tool (BLASTP, NCBI) analysis. The presence of SGI1 was also determined by PCR using U7-L12 and Lj-R1 primers specific for SGI1 left junction amplification [13].

### Pulsed-Field Gel Electrophoresis (PFGE)

To identify simultaneous infections by different *Salmonella* strains in a pig, the strains isolated from each animal showing identical phenotypic (i.e. serotype and AR) and AR genotypic (i.e. IC1 and SGI1) characteristics, were analysed by PFGE, following the Pulse-Net protocol described by the Centre for Disease Control and Prevention [20]. Briefly, the agarose plugs containing DNA were digested with 30 U XbaI (New England Biolabs). DNA fragments were separated (14**°**C, 18 h, 200 V) in 1% agarose gels with 0.5X Tris borate EDTA buffer, using a Cheff-DR II System (BioRad), and DNA was stained with 5% aqueous ethidium bromide solution. Lambda Ladder (BioRad) was used as molecular weight marker, and the DNA obtained from serotype Braenderup was used as control. *Salmonella* strains showing less than 95% PFGE profile similarity were considered as different.

## Results

Eight different serotypes were identified among the 140 *Salmonella* strains obtained. As shown in Table 1, serotypes Typhimurium, Rissen, Derby, and Kapemba were the most frequently isolated (46, 31, 20, and 16 strains, respectively) and widely distributed (in 8, 4, 2, and 3 animals, respectively). Fifty per cent of the pigs analysed (codes 1-7, Table 1) were found infected simultaneously by different *Salmonella* strains, since 2 or 3 different serotypes were identified in each pig. Typhimurium was the serotype most frequently isolated (6 out 7) in these 7 pigs. Interestingly, serovar Kapemba was always found simultaneously with Typhimurium. The remaining 7 pigs were found infected with a single serotype.

**Table 1:** **Serotypes and antimicrobial resistance (AR) of *Salmonella *** **strains isolated from fattening pigs mesenteric lymph nodes**^**a**^

**Animal code**	**Serotypes**	**AR profile**^**b**^	**Class 1 integron size/genes**^**c**^	**Total no. of strains isolated/pig**
	**(No. of colonies)**	**(No. of colonies)**	**(No. of colonies)**	
1	Typhimurium (4);	ACSSuTNx (4);	2000 bp/*blaoxa*30-*aadA1* (4);	2
	Kapemba (6)	CSSuT (6)	1000 bp/*aadA1* (6)	
2	Typhimurium (8);	ACSSuT (8);	2000 bp/*blaoxa*30-*aadA1* (8);	2
	Kapemba (2)	CSSuT (2)	1000 bp/*aadA1* (2)	
3	Typhimurium (1);	ACSSuT (1);	2000 bp/*blaoxa*30-*aadA1* (1);	3
	Kapemba (8);	CSSuT (8);	1000 bp/*aadA1* (8);	
	subsp*. arizonae* 48: z4,z23:- (1)	Susceptible (1)	None (1)	
4	Typhimurium (2);	ACSSuT (2);	2000 bp/*blaoxa*30-*aadA1* (2);	2
	subsp. *enterica* 6,7:-:1,5^d^ (8)	SSu (8)	None (8)	
5	Typhimurium (3);	ACSSuT (3);	2000 bp/*blaoxa*30-*aadA1* (3);	2
	Goldcoast (7)	ACST (7)	2000 bp/*drfA12-aadA2* (7)	
6	Typhimurium (8);	ACSSuT (8);	1000+1200 bp/*aadA2-pse1* (8)^e^;	2
	Rissen (2)	T (2)	None (2)	
7	Rissen (9);	ASSu (9);	2000 bp/*drfA12-aadA2* (9);	2
	Subsp*. arizonae* 48:z4,z23:- (1)	Susceptible (1)	None (1)	
8	Rissen (10)	ACST (3); A (5); Susceptible (2)	None (10)	3
9	Rissen (10)	ASSu (5); SSu (5)	2000 bp/*drfA12-aadA2* (10)	2
10	Derby (10)	SSuT (8); T (2)	None (10)	2
11	Derby (10)	SuT (5); T (5)	1000 bp/*aadA1* (5); none (5)	2
12	Typhimurium (10)	ACSSuTNx (10)	1000+1200 bp/*aadA2-pse1* (10)^e^	1
13	Typhimurium (10)	ACSSuT (10)	2000 bp/*blaoxa*30-*aadA1* (10)	1
14	Bredeney (10)	SuTNx (10)	2000 bp/*drfA12-aadA2* (10)	1
Total	8 (140)	12	3 amplicon size/ 4 IC1 types	NA

^a^A total of 140 CFU (10 CFU/pig) isolated in selective BGA or XLD media were purified in agar and characterized; ^b^Antimicrobial agents showing AR strains: (A) Ampicillin and/or Amoxicillin+Clavulanic acid; (C) Chloramphenicol; (S) Streptomycin and/or Gentamicin; (Su) Sulfonamides and/or Trimetoprim+Sulphometoxazole; (T) Tetracycline and/or Doxicycline; (Nx) Nalidixic acid; ^c^Genes identified by IC1 amplicons sequencing; ^d^Flagellar antigen phase 2 was not detected; ^e^SGI1 was detected; NA: Not Applicable.

Regarding phenotypic AR characteristics, pigs were infected with at least one multi-AR strain, and a total of 12 different AR profiles were identified (Table 1). The AR profile most frequently identified (46 strains) was ACSSuT, with or without additional resistance to Nx (Table 1). Genotypically, strains showing 3 types of IC1 were identified in 12 pigs (85.7%), showing amplicons of either 1000 bp (21 strains from 4 pigs), 2000 bp (64 strains from 9 pigs), or a double band of 1000 bp and 1200 bp each (18 strains from 2 pigs) (Table 1). These IC1 were absent in the 4 strains found susceptible to all antimicrobials as well as in the other 33 strains showing AR to one (aminopenicillins or tetracyclines) or several (SSu, SSuT or ACST) agents.

Amplicon sequencing allowed the identification of IC1 carrying 4 different AR gene cassettes: (i) *blaoxa*30-*aadA1* contained in 2000 bp amplicons of Typhimurium strains; (ii) *drfA12-aadA2* contained in 2000 bp amplicons of Goldcoast, Rissen and Bredeney; (iii) *aadA1* contained in 1000 bp amplicons of Kapemba and Derby; and (iv) *aadA2-pse1* contained in 1000 plus 1200 bp amplicons (Table 1). This latter IC1 was found only in Typhimurium strains, and associated with both the ACSSuT penta-AR profile and the presence of SGI1. Accordingly, these strains showed the characteristics of the DT104 phage-type. The remaining 28 Typhimurium strains (from 6 pigs) also showed the ACSSuT penta-AR profile but only the 2000 bp *blaoxa*30-*aadA1* IC1 amplicon not associated with SGI1 was amplified (Table 1). Similarly, the 16 Kapemba strains (found in 3 pigs) were resistant to CSSuT and carried a single 1000 bp IC1 containing the *aadA1* AR gene (animal codes 1-3, Table 1). Interestingly, in 4 out of the 7 pigs infected with a unique *Salmonella* serotype (animal codes 8-11, Table 1), 2 or 3 different AR profiles were identified, regardless of the presence/absence and size/sequence of IC1 amplicons. This clearly indicates also the presence of different *Salmonella* strains infecting the same animal.

The remaining 3 pigs (animal codes 12-14, Table 1) were infected by a unique and homogeneous *Salmonella* strain, as confirmed by PFGE. Overall, 11 out of the 14 pigs studied were infected simultaneously by at least 2 different *Salmonella* strains.

## Discussion

To the best of our knowledge, this is the first report in pigs demonstrating that the same animal may be naturally infected by multiple *Salmonella* strains. For this, a thorough microbiological analysis of MLN was carried out in a limited number of animals belonging to farms with high salmonellosis prevalence and where multiple circulating *Salmonella* strain types were previously identified. Although it was not the objective of this study, our results suggest that *Salmonella* co-infections may be quite common in pig herds with multiple *Salmonella* circulating strains.

The existence of multiple infections in the same animal suggests that pigs can be either infected simultaneously during a brief period either through one or multiple sources (i.e. food, water, environment, etc.) or re-infected along the different stages of their productive life (i.e. postweaning, growing, and finishing periods). The possibility of re-infection has been previously proposed in sows from which different *Salmonella* serotypes were isolated from faecal samples collected at different time points [21]. Nevertheless, the presence of the pathogen in faeces does not necessarily mean an active infection, as *Salmonella* can circulate passively through the animal’s gut lumen. Faecal culture results should interpreted with caution since these samples can also be easily cross-contaminated during collection. In our study, however, the presence of *Salmonella* in MLN would reflect a true infection. In the present study, the possibility of MLN cross-contamination was very limited because (i) sampling was performed at different dates; (ii) we used single-use gloves and clothes, liquid disinfectant (DD445, A&B Laboratorios de Biotecnología) and sterilized instruments each time; (iii) MLN samples were individually collected in sterile plastic bags; and (iv) once in the laboratory, MLN samples were defatted and externally decontaminated through alcohol immersion and flaming, as recommended by the ISO method [8]. Thus, our results demonstrate the presence of active multiple infections as different *Salmonella* strains were isolated from MLN tissue, which could be colonised only after active enterocyte invasion [5].

Typhimurium and Rissen were the most prevalent *Salmonella* serotypes identified, which is in agreement with the findings of a large study performed previously in the same pig population [8]. It is worth to note that Kapemba was also found in a relative high frequency (11.4%) but always accompanied by Typhimurium. In contrast, Kapemba was rarely isolated at both individual (1.8%) and herd (3.7%) levels in the previous large study [8], and also in the baseline study carried out in the EU [5]. Such differences could be due to the different identification strategy used in these studies, since serotyping was performed exclusively on one colony from each animal in these large-scale studies.

For epidemiological purposes, the international standards recommend confirming the presence of *Salmonella* by typing one (up to 5) colony per sample [14]. Although this microbiological approach may be useful to confirm infection, it could easily overlook the presence of the less predominant strains, since the more prevalent ones appear to be always present in MLN co-infections (Table 1). Therefore, epidemiological studies based on the serotyping of a single bacterial colony, such as those focused on the eradication of specific serotypes (i.e. national control programmes against major zoonotic *Salmonella* serotypes) may be overrepresenting the prevalent strains and understimating other potentially pathogenic but less predominant serotypes. Likewise, outbreak investigations would require the analysis of several colonies from the same animal to identify the main source of infection. Systematic screening of multiple colonies from individual pig samples could contribute to the trace back of many *Salmonella* outbreaks origin in humans [22].

The coexistence of *Salmonella* strains with different multi-AR profiles within the same pig as primary reservoir may have important epidemiological consequences. This can promote exchange and propagation of mobile genetic elements between bacterial strains that share the same biological niche *in vivo*. In this study, co-infections by *Salmonella* strains showing different AR profiles were relatively frequent, regardless of the serotype. In fact, most of animals studied (11 out 14) were simultaneously infected by strains showing 2 or 3 different AR profiles. The finding that pigs with co-infections showed different AR profiles against common antimicrobial agents suggested that genetic exchange could be taking place within the same animal, generating a genetic variability in *Salmonella*. Horizontal transfer of AR genes or IC1 was not observed in three animals (animal codes 3, 7 and 8, Table 1) harbouring both susceptible and multi-AR strains, but genetic exchanges could not be excluded in these animals [23].

SGI1 was detected only in Typhimurium strains from two animals (animal codes 6 and 12, Table 1) containing also the characteristic IC1 1000-1200 bp double band with the double *aadA2*-*pse1* gene cassette, and the typical penta-AR (ACCSuT or ACSSuTNx) of DT104 phagetype [13]. The widespread dissemination of Typhimurium DT104 clone was particularly relevant since it was first isolated in the early 80´s in UK cattle and subsequently reported worldwide in a wide variety of animal species including pigs, animal foodstuff, and humans [24,25]. Similarly, other emergent variants, such as the monophasic variant of Typhimurium DT193 phagetype carrying the multi-AR ASSuT [26] have been detected, and epidemiological surveillance is therefore recommended [27].

IC1 genotypes are the most frequent carriers of AR genes in *Salmonellae*, but these genes could also be present in other integrons [28,29]. In fact, IC1 was not detected in some strains resistant to one (aminopenicillins or tetracycline) or more (SSu, SSuT or ACST) antimicrobial agents. However, a quick detection of AR strains is critical for a successful treatment in human beings. Thus, the IC1 PCR analysis of several *Salmonella* colonies from a *Salmonella*-positive sample should be considered as a suitable (quick, easy, low cost, and effective) screening approach for detecting multi-AR genetic mobile elements.

The presence of simultaneous infections by *Salmonella* strains of different serotype, serogroup and AR profiles could also have immunological implications on the host-pathogen interaction. Thus, if infections occur over time, our results may suggest a limited Genus-, serogroup- and species- specific protection of pigs after a primary *Salmonella* infection, but further studies are needed for a better understanding of the host-pathogen interactions. The existence of co-infections in a single animal and within the same herd may assist in the development of effective vaccines, therapeutics and control programmes against pig salmonellosis.

## Conclusions

This study demonstrates the presence of simultaneous infections by different *Salmonella* strains in asymptomatic pigs. Systematic screening for multiple strains from individual MLN samples is a time-consuming strategy not routinely applied in laboratory protocols but essential to understanding both the pathogenesis and epidemiology of *Salmonella* infections in pigs. It may also be useful to trace back the origin of salmonellosis outbreaks in humans. Further studies in larger pig populations should be carried out to confirm that *Salmonella* co-infections are a common event in swine.

## Competing interest

The authors declare that they have no competing interests.

## Authors’ contributions

All authors participated in the draft of the manuscript. Moreover, VG, BSR, SS and AZB carried out the sampling collection in slaughterhouses, microbiological isolation, biochemical identification, antimicrobial resistance characterization, and PFGE studies; YDT and CDF carried out the serotyping; RCMJ participated in the design of the study and discussion of results; and MJG conceived, designed, and coordinated the study, and wrote the final manuscript. All authors read and approved the final manuscript.
